# Basic Mechanisms of JAK Inhibition

**DOI:** 10.31138/mjr.31.1.100

**Published:** 2020-06-11

**Authors:** Chung MA Lin, Faye AH Cooles, John D. Isaacs

**Affiliations:** Translational and Clinical Research Institute, Newcastle University and Musculoskeletal Unit, Newcastle upon Tyne Hospitals NHS Trust, Newcastle upon Tyne, United Kingdom

**Keywords:** JAK inhibitors, disease-modifying drugs, rheumatoid arthritis

## INTRODUCTION

Over recent decades, treatment options for inflammatory diseases such as rheumatoid arthritis (RA) have increased dramatically. These range from orally available steroids and conventional synthetic disease modifying drugs (csDMARDs) to parenteral biological therapies (bDMARDs). Most recently, with advances in our understanding of cell signalling pathways, we can target small molecules associated with intracellular signal transduction.^1^ These orally available drugs form a new category of treatment known as targeted synthetic DMARDs (tsDMARDs).^2^ The first drug class within this category to gain marketing authorisations are the Janus Kinase inhibitors (JAK inhibitors or jakinibs).^3^ By inhibiting Janus Kinases, these drugs inhibit signalling through a variety of cytokine and haematopoietic growth factor receptors.^4^

There are four members of the JAK family, and all are receptor-associated tyrosine kinases (JAK1, JAK2, JAK3 and TYK2).^4^ Tyrosine kinases are phosphotransferase enzymes which transphosphorylate tyrosine residues on other proteins. This process can trigger (usually) or hinder (less commonly) the activity of the target protein, often as part of an enzymatic cascade.^5,6^ All JAKs work in a similar manner, usually in association with type I and II cytokine receptors, which are intrinsic elements of immune responses.^7,8^ Consequently, inhibiting these enzymes has great potential for controlling unwanted or overactive immune pathways.^9^ It is important to understand both the role of cytokines in regulating immune function and the JAK-STAT (signal transducer and activators of transcription) pathways in order to fully appreciate the true value of JAK inhibition, especially in relation to its role in diseases such as RA.^5,8^

Cytokines form a large family of (mostly) soluble mediators, which are responsible for controlling a wide range of bodily processes, from growth to haematopoiesis. They play an important role in both innate and adaptive arms of the immune response.^9,10^ Unsurprisingly, an imbalance of their activity is associated with a number of different autoimmune diseases and malignancies.^11,12^ Anti-inflammatory agents such as glucocorticoids, as well as csDMARDs, can impair cytokine secretion and downstream activities, but long-term use and off-target effects result in unwanted side effects, such as osteopenia and liver toxicity.^13,14^ Some bDMARDs (TNF inhibitors, IL-6 receptor blockers) target pro-inflammatory cytokines themselves, with significant benefit. These bDMARDs have contributed to the revolution in the management of autoimmunity but they are expensive, require parenteral administration, as well as co-prescription with methotrexate (MTX) to achieve optimal outcomes.^15–17^

Many patients with RA prefer oral drug therapy, triggering an unmet need for potent oral medications.^17^ Using synthetic, orally available drugs to target intracellular signalling pathways has the potential to meet this need, potentially matching biological efficacy within a pill.^2–6^ The JAK-STAT pathways provided rational targets due to their involvement in cytokine signalling, including cytokines thought to be active in RA, such as interleukins, interferons and growth factors.^5,8^

## JAK-STAT PATHWAY

**Figure 1.**
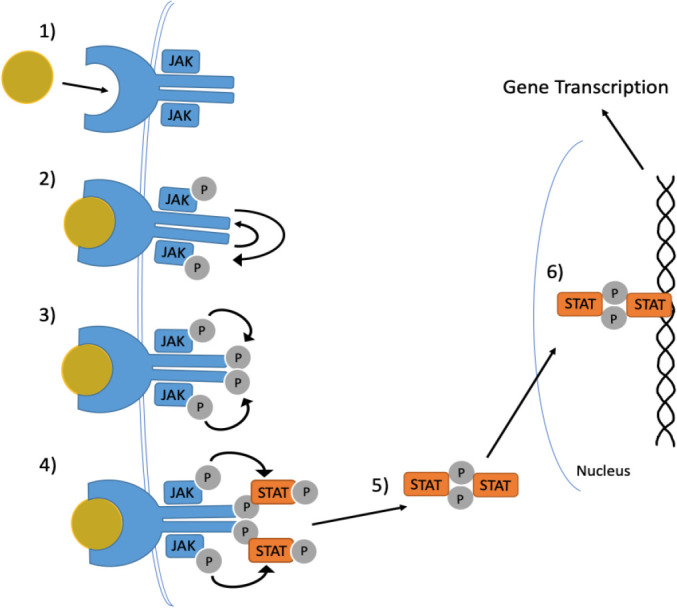
The JAK-STAT pathway. Step 1) The ligand (usually a cytokine) binds and cross-links its receptor. Step 2) The associated JAKs transphosphorylate and activate each other. Step 3) The activated JAKs phosphorylate the receptor tail. Step 4) The receptor tail becomes a docking site for recruited STAT proteins, which themselves are phosphorylated by the activated JAKs. Step 5) The phosphorylated STATs dissociate from the receptor and dimerise. Step 6) STAT dimers translocate to the nucleus where they regulate gene transcription. JAK = Janus kinase, P = phosphate group, STAT = signal transducer and activator of transcription.

JAK-STAT pathways are utilised by type I and II cytokine receptors, as well as by receptors for interferons and growth factors. These receptors lack intrinsic catalytic activity and rely on JAKs for downstream responses and subsequent modulation of gene expression (*Figure 1*).^1^ Janus is the Greek god of doorways, looking both outside and inside a room, and illustrates how JAKs facilitate signals from the cell surface into the cell.^18^ Each cytokine receptor is paired with a different JAK pair, usually as heterodimers. Upon cross-linking by its cytokine, the receptor-associated JAKs transphosphorylate one another. The activated JAKs in turn phosphorylate the cytokine receptor tail. The phosphorylated receptor forms a docking site for STATs, that otherwise reside in the cytosol. These STATs are then phosphorylated by the JAKs before dissociating from the receptor and themselves forming heterodimers or homodimers. They then translocate to the nucleus where they act as transcription factors, regulating gene expression. ^19^ There are seven mammalian STATS which, like JAKs, associate with different signalling pathways.^8^

The importance of these pathways in health and disease has been demonstrated through multiple studies involving knockout mice and mutagenized cell lines.^20–22^ Of more relevance, certain types of human primary immunodeficiencies such as severe combined immunodeficiency (SCID), are caused by non-redundant mutations related to these pathways.^23,24^ In contrast, overexpression of these pathways is associated with both autoimmune disease and malignancy.^25–28^ Consequently, their blockade provides a means to block, simultaneously, the actions of multiple key cytokines associated with auto-immunity.^1^

## SPECIFICITY VS SELECTIVITY

Each JAK enzyme contains an ATP binding pocket which is critical to their function. It is the ATP bound within the pocket that supplies the phosphate group intrinsic to JAK activity. Whilst structurally similar, each JAK has a subtly different ATP binding pocket.^29^ It is worth adding that there are over 500 tyrosine kinases in the human genome, each of which has a related mode of action and possesses an ATP binding pocket.^1^ Drugs that inhibit these enzymes, such as the jakinibs, generally act by impeding ATP binding.^29,30^ Because ATP binding pockets differ between the JAKs (and more widely within the tyrosine kinase ‘superfamily’), it should be possible to find drugs that selectively block a particular JAK.^11^

**Figure 2.**
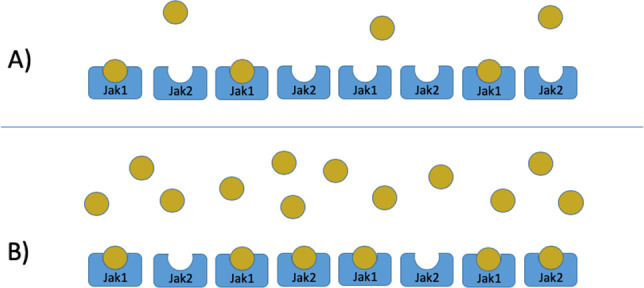
A simple demonstration of drug selectivity. A) At low concentration, JAK1 is more completely blocked due to preferential binding. B) As the concentration rises, JAK2 starts to become blocked but not as completely as JAK1, for which the affinity remains higher.

The selectivity of a JAK inhibitor is fundamentally different to the specificity of a biologic drug. Rheumatologists have become familiar with biologics which, as a consequence of nature’s highly evolved antibody design, are highly specific for their target (TNF, IL-6 receptor) with virtually no possibility for ‘off-target’ effects on other molecules or pathways.^31^ This is sometimes referred to as a lock and key mechanism of action – most keys simply do not work in the ‘wrong’ lock.^32^ In contrast, small molecule enzyme inhibitors, such as the jakinibs, are in a Michaelis-Menten equilibrium with their substrate and ATP.^33^ A highly selective JAK inhibitor (eg, with selectivity for JAK1), will compete with ATP on JAK1 with a higher potency than on JAK2, JAK3 or TYK2. However, as the intracellular concentration of the drug increases it is likely to affect ATP binding to these other JAK family members, with loss of selectivity (*Figure 2*).^33,34^ Rather than lock and key, this can be thought of as fingers in gloves, and it is unlikely that a jakinib can be developed that is completely specific for a single JAK.^35^ Intracellular concentration depends not only on dosing, but on factors specific to each patient, such as age, weight, liver and kidney function, other medications, etc. It should also be stressed that selectivity is usually deduced from reductionist laboratory enzymatic or cellular assays, which may or may not reflect the in vivo situation.^12^ Early phase (phase 1, 2) clinical trials aim to identify the optimal drug dose, at a population level, in terms of achieving optimal selectivity.^36^ However, it is real life experience, in a typical patient population, when rheumatologists need to judge the selectivity of a particular therapeutic.

## WHICH PATHWAY TO BLOCK?

**Figure 3.**
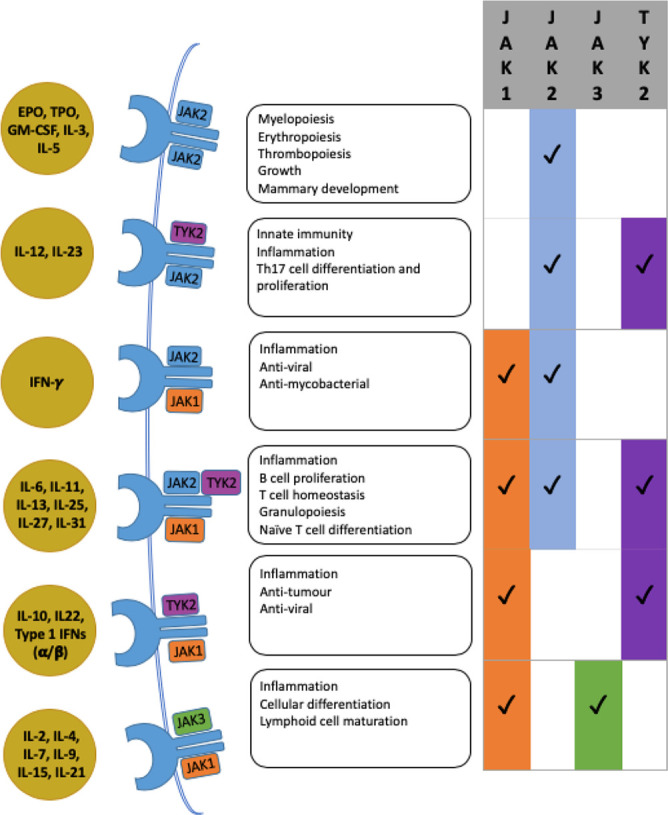
Different JAK combinations with their subsequent downstream effects, each mediated by a specific subset of cytokines.^5,6,40,45,46^

At the time of writing, there are already three licensed jakinibs, with several more in clinical trials.^4^ In terms of which JAK provides the optimal therapeutic target, the decision is complex, not least because of heterodimeric JAK pairing. Only JAK2 acts as a homodimer, in terms of haematopoietic growth factor signalling (*Figure 3*).^37^ Most JAKs illustrate significant redundancy, being involved in several pathways.^4^ In contrast, JAK3 only transduces signals from γ-chain cytokines IL-2, -4, -7, -9, -15 and -21.^38,39^ These play a central role in the adaptive immune response, with certain cytokine deficiencies resulting in a SCID phenotype. Human JAK3 deficiencies and inactivating mutations also result in SCID.^21–24^ The fact that therapeutic blockade of JAK3 is achievable without life-threatening toxicity relates back to the reversible and/or transient enzymatic blockade intrinsic to small molecule drugs versus the permanent absence with genetic deficiencies. It follows from the above that a drug that is selective for JAK3 should have relatively defined downstream effects, predominantly reducing the activity of γ-chain cytokines.^21–24^

In contrast to JAK3, the other JAK family members play a more redundant role, featuring in several pathways (*Figure 3*). These include important pro-inflammatory cytokines such as type I interferons (IFN-I) and IL-6, as well as IL-12, -23 and IFN-γ.^40^ Hence, inhibition of these pathways will have relatively pleiotropic effects. This is not necessarily undesirable but will reduce the differences between drugs which, at least in vitro, are differentially selective. Of note, the haematopoietic growth factors erythropoietin (EPO), thrombopoietin (TPO), granulocyte macrophage colony stimulating factor (GM-CSF), IL-3 and IL-5 signal via JAK2 homodimers.^41^ Blocking this pathway theoretically could cause anaemia, leukopenia and thrombocytopenia and, arguably, may be a pathway to avoid.^8^ Nonetheless, GM-CSF plays a pro-inflammatory role in RA, whose blockade has been shown to be beneficial.^4^ Whether a jakinib could have differential selectivity for a JAK2 homodimer versus a heterodimer is uncertain, but certainly JAK2 inhibition is not universally associated with haematological side effects. In humans, genetic deficiencies in JAK1 and JAK2 are not recognised, consistent with knockout mice studies showing perinatal and embryonic lethality respectively.^42–44^ Conversely, human TYK2 deficiencies result in an impaired immune response against viral and bacterial pathogens, likely due to defects in the transduction of IFN-I, IL-12 and IL-23 signalling.^21^ As mentioned above, transient and generally reversible therapeutic targeting of these pathways does not reproduce these serious genetic defects.

## JAK INHIBITORS IN PRACTICE

Tofacitinib, baricitinib and, most recently upadacitinib, are each approved for the treatment of RA. In terms of selectivity, tofacitinib is more selective for JAK 1, 2 and 3 versus TYK2. Baricitinib is more selective for JAK 1 and 2, and upadacitinib for JAK1.^4^ All are of proven efficacy in the management of RA. According to *Figure 3*, tofacitinib and baricitinib might be expected to have similar activity in terms of cytokine blockade, with upadacitinib having less effect on haematopoietic growth factor signalling and IL-12/23 signalling.^4^ In contrast, recent in vitro studies using human peripheral blood mononuclear cells suggest that, whilst quantitative differences exist in the potency of these jakinibs to inhibit cytokine signalling, these differences are perhaps less than expected and not always in the expected order of potency.^47^ Similarly, clinical trials have not revealed major differences in efficacy or safety between these three agents in terms of efficacy or adverse events.^4^ Clinical trial populations are, of course, quite tightly selected in terms of factors such as renal and liver function and lack of serious comorbidities, and it remains possible that real life pharmacokinetic factors may reveal differences between these drugs in certain populations.^48^

## CONCLUSION

There have been great advances in the pharmacological management of RA and other autoimmune diseases over recent decades, and jakinibs are emerging as a new therapeutic option. As small molecule, chemically synthesised drugs their advantages include oral administration, and reduced manufacturing costs compared with biologics.^49–51^ Clinical trials also suggest efficacy with or without concomitant MTX administration. Their short half-lives should equate with briefer peri-operative interruption compared to biologics and, potentially, more rapid reversal of adverse effects.^49^ Most importantly they demonstrate efficacy at least equivalent to biologics, with a similar profile of adverse events.

Despite these advantages, some of the major cytokines involved in RA pathogenesis, specifically TNF-α, IL-1 and IL-17, are not dependent on JAKs for their signalling.^52–54^ Whether or not combination therapy, for example with a jakinib and TNF inhibitor, will show benefit over a jakinib or TNF inhibitor alone awaits appropriate testing. However, the chronic inflammatory microenvironment is complex,^55^ and indirect inhibition of these cytokines remains possible in association with jakinib therapy.

The field of JAK inhibition remains in its infancy. Several other jakinibs are in development, and trials of the approved drugs are ongoing in a range of immune mediated inflammatory diseases. As suggested elsewhere in this brief review, real world evidence will be particularly important in defining differences between the jakinibs, particularly in terms of their adverse event profiles. Nonetheless, the evidence to date illustrates that jakinibs are likely to provide a potent therapeutic option in a range of autoimmune and inflammatory diseases.
